# Auditory Electrophysiological Findings in Children with Developmental Language Disorder: A Systematic Review

**DOI:** 10.3390/diagnostics16132090

**Published:** 2026-07-03

**Authors:** Diego Lourenço dos Santos Silva, Dandara Felipini, Piotr Henryk Skarzynski, Caroline Donadon, Milaine Dominici Sanfins

**Affiliations:** 1 Department of Speech-Language-Hearing Pathology, Universidade Federal de São Paulo–Escola Paulista de Medicina (UNIFESP), São Paulo 04044-020, Brazil; dsilva12@unifesp.br (D.L.d.S.S.); felipini.dandara@unifesp.br (D.F.); 2 Department of Teleaudiology and Screening, World Hearing Center, Institute of Physiology and Pathology of Hearing, 05-830 Warsaw, Poland; 3 Department of Otolaryngology, Institute of Sensory Organs, 05-830 Warsaw, Poland; 4 Independent Researcher, Campinas 13293-266, Brazil; caroldonadon.fono@gmail.com

**Keywords:** children, adolescent, language development disorders, auditory evoked potential, electrophysiology

## Abstract

**Background/Objectives:** Developmental Language Disorder (DLD) is a persistent neurodevelopmental condition with an estimated prevalence of 7% in preschoolers. It is characterized by significant impairments in language acquisition and use, in the absence of an identified biomedical cause. The potential link between DLD and central auditory processing has encouraged the investigation of auditory evoked potentials as research tools; however, the existing literature remains notably dispersed and heterogeneous. To systematically synthesize evidence concerning auditory electrophysiological findings in children with DLD. **Methods:** Systematic searches were conducted in PubMed, Web of Science, and Scopus for studies published between January 2016 and March 2026. Keywords were combined using AND/OR operators. Two independent reviewers performed screening and data extraction, with discrepancies resolved through consensus. Risk of bias was assessed using the JBI tool for analytical cross-sectional studies. A structured narrative synthesis (SWiM) was applied to the findings. **Results:** Seven studies were included, with a combined total of 480 participants across all enrolled samples (including DLD, control, and other clinical subgroups), aged 2 years and 11 months to 10 years. The Frequency Following Response (FFR) appeared to show greater sensitivity, with alterations in both temporal components (waves C and D) and spectral components (F0 and F2), particularly under noise conditions. Findings for click-ABR were inconsistent across studies, suggesting limited sensitivity in cases of isolated DLD. Long-latency auditory evoked potentials (N2 and P300) exhibited prolonged latencies, potentially reflecting cortical immaturity and impaired attentional discrimination. The N400 potential suggested delayed or atypical semantic processing in a single investigation. **Conclusions:** The available evidence points toward a pattern of impairment in individuals with DLD that requires cautious interpretation, potentially encompassing subcortical, cortical, and linguistic encoding. Methodological heterogeneity across studies, combined with the absence of adolescent samples, highlights significant gaps in the current research regarding electrophysiology and DLD. FFR and Long-Latency Auditory Evoked Potentials (LLAEP/P300) assessments may warrant further investigation as auxiliary electrophysiological measures for the characterization of DLD, pending replication in larger and more homogeneous samples.

## 1. Introduction

Developmental Language Disorder (DLD) historically referred to in the international literature as Specific Language Impairment (SLI) is recognized as a persistent neurodevelopmental condition. It is characterized by significant impairments in language acquisition and use in the absence of identifiable biomedical causes, such as sensory deficits, neurological conditions, or environmental deprivation [1,2]. These manifestations may involve phonological, morphosyntactic, semantic, and pragmatic domains across both expressive and receptive modalities, often persisting throughout the lifespan [2]. Epidemiologically, DLD is considered the most prevalent neurodevelopmental communication disorder. Estimates suggest a prevalence of approximately 7% to 7.58% in children aged 5 to 6 years, according to the British SCALES population study [3], 6.4% at age 10 in the Australian Raine cohort [4], and 8.5% among Mandarin-speaking preschoolers in Shanghai [5].

A recent systematic review on prevalence (Hill et al., 2024) corroborates this cross-cultural convergence while identifying the heterogeneity of diagnostic criteria as a primary source of variation between studies [6]. The functional impact of DLD is substantial; research indicates that approximately 88% of children with the disorder fail to meet curriculum standards in their first school year [3]. Furthermore, DLD is associated with persistent socio-emotional challenges [7] and typically extends into adolescence and adulthood, potentially affecting academic achievement, professional performance, and overall quality of life [8,9].

It is pertinent to note that the consolidation of the term “DLD” is a relatively recent development. For decades, language impairments without an identifiable biomedical etiology were described using a variety of labels including developmental dysphasia, language delay, and specific language impairment (SLI) often with divergent diagnostic criteria, particularly regarding the requirement for a discrepancy between nonverbal IQ and linguistic performance [2,3,10]. In 2016, the CATALISE project, led by Bishop and colleagues, sought to establish a consensus among experts concerning diagnostic criteria and terminology [10]. This first phase identified the features characterizing language impairments in children and recommended discontinuing the use of discrepancy-based criteria. Subsequently, the 2017 CATALISE-2 consensus [2] formally recommended the adoption of the term “Developmental Language Disorder” (DLD) and established a two-stage diagnostic process: (i) the identification of persistent language impairments with functional impact and poor prognosis, in the absence of compromised learning opportunities; and (ii) the verification of co-occurring biomedical conditions. When such conditions are present (e.g., autism spectrum disorder, genetic syndromes), the presentation is instead termed a “language disorder associated with” the specific condition.

Individuals with DLD appear to exhibit a significant dependence on auditory information processing. This may be explained by the fact that language emerges from the complex interaction between the maturation of neuro-auditory-linguistic networks and early exposure to oral language during critical windows of plasticity [11,12]. The “Rapid Temporal Processing” hypothesis [13,14] postulates that children with DLD face challenges in processing brief auditory information or signals presented in rapid succession. While behavioral auditory processing tests are traditionally used to assess these skills, their sensitivity may be limited by factors such as attention, motivation, and cooperation [15,16]. Consequently, electrophysiological measures have emerged as a potentially robust alternative for the objective analysis of auditory information processing [16].

Electrophysiological measures of hearing, also known as Auditory Evoked Potentials (AEPs), represent synchronized neural activity generated in response to acoustic stimuli. These are organized into a functional hierarchy that maps the signal trajectory from the cochlea to the cortex and associated cognitive circuits [16,17,18]. Since each potential reflects a specific aspect of auditory processing, they may serve as sensitive tools for monitoring neurodevelopmental dysfunctions, such as those observed in DLD. To date, no systematic review has integrated findings across the full hierarchy of auditory evoked potentials from brainstem to cortex in children with DLD using a GRADE-based appraisal of evidence certainty, representing a gap that the present review seeks to address.

Given the relevance of auditory electrophysiology in neurodevelopmental contexts, the present study provides a systematic review of the literature. The primary objective is to analyze the available evidence regarding the relationship between Auditory Evoked Potentials and Developmental Language Disorder in children and adolescents aged 1 to 12 years; however, as detailed in the Results and Limitations, none of the studies meeting the eligibility criteria enrolled participants beyond 10 years of age, such that the evidence synthesized here pertains specifically to the pediatric population up to that age.

## 2. Methods

This systematic review was conducted in accordance with established guidelines (PRISMA 2020) [19] for study identification, selection, eligibility, and inclusion (Supplementary Materials, Table S1). The research question was formulated using the PICOT strategy, encompassing the following elements: Population (P), Intervention (I), Comparison (C), and Outcome (O).

As previously noted, the nomenclature used to describe language impairments without a defined etiology has undergone significant shifts over the last decade. Following the 2016 consensus, the term Developmental Language Disorder (DLD) began to replace Specific Language Impairment (SLI), consolidating a standard for the diagnosis. Consequently, studies published prior to 2018 may still employ the former terminology, as the adoption of the current nomenclature occurred gradually during that transitional period. Table 1 details the research question and its constituent elements according to the PICO strategy.

### 2.1. Eligibility Criteria

The study selection process was guided by the following inclusion criteria:

1. Investigations focused on auditory electrophysiological assessments;
2. Participants aged between 1 and 12 years, encompassing toddlers, preschoolers, and school-aged children;
3. A formal and confirmed diagnosis of Developmental Language Disorder (DLD);
4. Peripheral hearing sensitivity within normal clinical limits, as verified by pure-tone audiometry, speech audiometry, and immittance testing;
5. Electrophysiological evaluations specifically utilizing auditory stimuli;
6. Peer-reviewed articles published within the last 10 years (2016–2026).

The following exclusion criteria were applied:

1. Studies in which the entire DLD sample presented confirmed co-occurring neurodevelopmental disorders (e.g., Autism Spectrum Disorder, ADHD, or Intellectual Disability), precluding the characterization of isolated DLD. Studies that included additional clinical comparison subgroups alongside a distinct DLD subgroup, or that compared multiple diagnostic categories without a requirement for DLD participants to be free of comorbidities, were not excluded by this criterion;
2. Grey literature, including case reports, editorials, conference proceedings, and preprint manuscripts not yet submitted to peer review;
3. Animal models.

### 2.2. Search Strategy

A comprehensive and systematic search was conducted between February and March 2026. The search strategy was developed based on the PICO framework and implemented across PubMed, Web of Science, and Scopus databases. A combination of Medical Subject Headings (MeSH) and Health Sciences Descriptors (DeCS) was utilized, including the following terms in English: “Developmental Language Disorder”, “DLD”, “Specific Language Impairment”, “SLI”, “Language Disorders”, “Language Impairment”, “Evoked Potentials, Auditory”, “Auditory Evoked Potentials”, “Auditory Brainstem Response”, “ABR”, “BAEP”, “Frequency-Following Response”, “FFR”, “Event-Related Potentials”, “P300”, “LLAEP”, “Late Auditory Evoked Potentials”, and “Cortical Auditory Evoked Potentials”. These descriptors were systematically integrated using the Boolean operators AND and OR. Additionally, a manual screening of the reference lists of the selected articles was performed to identify further relevant investigations. The detailed search strategy is presented in Table 2.

### 2.3. Study Selection Process

The records identified across the three databases were imported into the Rayyan online platform (https://www.rayyan.ai/ (free version)) (Qatar Computing Research Institute—QCRI), where automated duplicate identification was performed and the blind review mode was activated to ensure independent screening. Subsequently, a blind screening of titles and abstracts was conducted to categorize studies for full-text review. Reasons for exclusion were systematically documented within the platform’s commentary tool. Potentially relevant articles were then subjected to a comprehensive full-text evaluation based on the predefined eligibility criteria. The selection process was conducted independently by two reviewers (D.L. and D.F.), with any discrepancies resolved through deliberation and consensus with a third reviewer (C.D.).

### 2.4. Data Extraction and Synthesis

Data extraction from the included studies was performed in a standardized manner using a pre-designed spreadsheet. The following variables were collected: author and year of publication; sample characteristics (sample size, age range, mean age, and sex); electrophysiological procedures; complementary assessments; equipment; and the primary results and conclusions of the studies. These results are presented in Table 3 and Table 4. The technical parameters utilized for the examinations in each study are detailed in Table 5, comprising: stimulus duration, stimulus intensity, presentation rate, high-pass filter, low-pass filter, artifact rejection, polarity, number of sweeps, analysis window, and band-pass filter.

A structured narrative synthesis was chosen over a meta-analysis due to the identification of substantial methodological and statistical heterogeneity among the included studies. This variability was observed across diverse electrophysiological protocols, which exhibited significant variations in stimulus duration (ranging from 40 ms to 206 ms), presentation rates, and signal filtering configurations (with bands ranging from 30 Hz to 3000 Hz). Furthermore, discrepancies in clinical characterization and the age ranges of the samples (2 years and 11 months to 10 years), combined with the absence of reported dispersion measures and standardized quantitative data in several publications, informed this decision. Under these circumstances, a structured narrative synthesis (SWiM—Synthesis Without Meta-analysis) was considered the most appropriate approach to ensure the integrity of the findings’ interpretation, thereby avoiding the risks of bias inherent in the statistical grouping of non-homogeneous data. Outcomes were grouped by electrophysiological potential [click-ABR, FFR, LLAEP/P300, N400, Auditory Steady-State Response (ASSR)] as the principal unit of synthesis, and the direction of the group difference (DLD versus control) reported by each study was used as the standardized effect descriptor across studies. When a study assessed a given potential but did not report extractable quantitative results for it, that outcome was classified as missing data for that study and excluded from the directional synthesis for the corresponding potential, rather than being counted as evidence of no difference; such instances are explicitly identified in the Results.

## 3. Results

### 3.1. Study Selection

A total of 449 records were identified through the initial database searches (97 from PubMed, 154 from Web of Science, and 198 from Scopus). Following the removal of 123 duplicate entries, 326 unique records remained for further evaluation. These records underwent a preliminary screening of titles and abstracts based on the pre-established eligibility criteria. During this phase, 313 articles were deemed ineligible, resulting in 13 studies being selected for comprehensive full-text assessment.

**Figure 1:**
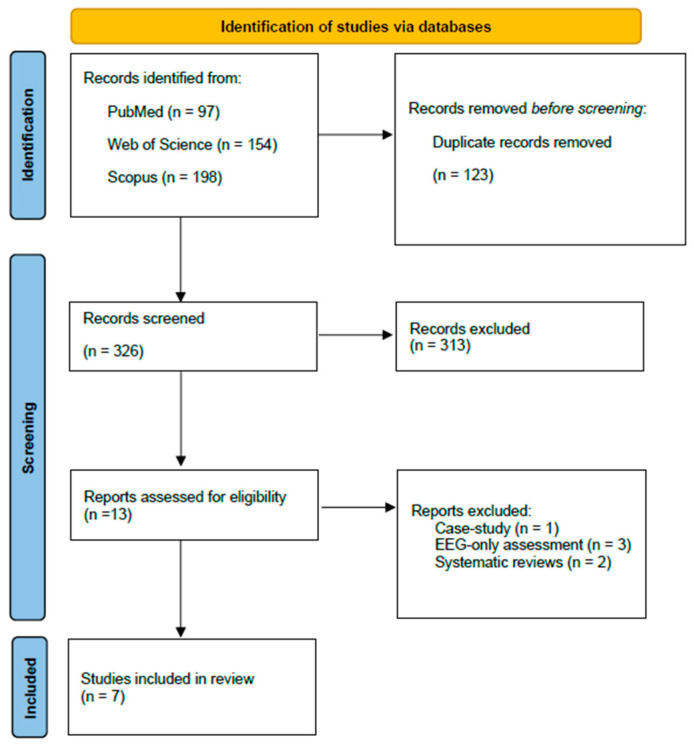
PRISMA 2020 [19] flow diagram for new systematic reviews which included searches of databases and registers only.

Upon thorough evaluation of these 13 articles, 6 were subsequently excluded for the following reasons: one was a case study, two were systematic reviews, and three focused exclusively on electroencephalography (EEG) for the proposed analysis. Ultimately, 7 studies met all inclusion criteria and were incorporated into this systematic review. The complete process of identification, screening, eligibility, and inclusion is illustrated in the PRISMA flow diagram (Figure 1). The PRISMA 2020 reporting checklist is provided as Supplementary Material (Table S1).

### 3.2. Methodological Quality Assessment (Risk of Bias)

The assessment of the methodological quality of the seven included studies, carried out using the JBI tool for analytical cross-sectional studies, demonstrated a predominance of moderate risk of bias. The methodological quality of the six analytical cross-sectional studies was appraised using the JBI Critical Appraisal Checklist for Analytical Cross-Sectional Studies (Table 6). Scores ranged from low-to-moderate to high risk of bias, with the main weaknesses concentrated in the identification and control of confounding variables (Q5–Q6).

Given that Aras et al. [26] did not include a typically developing control group and instead compared multiple diagnostic subgroups, this study was appraised separately using the JBI Critical Appraisal Checklist for Studies Reporting Prevalence Data (Table 7), which is better suited to its observational, non-comparative design.

### 3.3. Sample Characteristics

#### 3.3.1. Age

The age range of the participants in the included studies spanned from 2 years and 11 months to 10 years. Among the seven analyzed studies, two (28.6%) focused on the 7-to-10-year age group [22,23]. The remaining studies involved diverse age brackets, each represented by a single investigation (14.3%): 2 years and 11 months to 6 years and 6 months [26], 3 to 6 years [24], 3 to 7 years [20], 4 to 6 years [21], and 4 to 7 years [25].

#### 3.3.2. Sample Size—Control and Study Groups

Sample sizes varied significantly across the reviewed research. Three studies (42.9%) employed balanced cohorts with an equal number of participants in both the control and study groups: Gabr et al. [20] (*n* = 20), Wlodarczyk et al. [22] (*n* = 100), and Barman et al. [25] (*n* = 5). In contrast, Pijnacker et al. [21] reported a larger study group (*n* = 37) compared to the control group (*n* = 25), a pattern also observed in Elmahallawi et al. [24] (SG: *n* = 25; CG: *n* = 15). Conversely, Kwok et al. [23] utilized an inverse configuration, with a larger control group (*n* = 67) relative to children with DLD.

Notably, Aras et al. [26] focused exclusively on clinical cohorts. The participants were distributed according to their respective diagnoses: Developmental Language Disorder (*n* = 39), autism spectrum disorder (*n* = 16), articulatory pathology (*n* = 15), underlying organic brain pathology (*n* = 15), cognitive delay (*n* = 15), and speech and language impairments (*n* = 23). The authors opted for this design to analyze ABR results across different pathologies, aiming to identify potential correlations between electrophysiological findings and specific clinical conditions. This study was included in the present review as it featured a distinct subgroup with DLD.

#### 3.3.3. Sex

Regarding sex distribution, four studies did not report this variable within their methodology [21,23,25,26]. In the three studies that provided these data [20,22,24], the variables were analyzed in an aggregated manner. The Control Group (CG) comprised 135 participants, of whom 54 were male (40.0%) and 81 were female (60.0%), indicating a female predominance. In contrast, the Study Group (SG), totaling 145 participants, showed a male predominance, with 101 males (69.7%) and 44 females (30.3%). Consequently, an inverse distribution between groups was observed, with higher female representation in the typically developing group and higher male representation in children with DLD.

### 3.4. Language and Auditory Assessments

#### 3.4.1. Language Assessment

Regarding language evaluation, the Preschool Language Scale (PLS) was utilized in two of the seven studies [20,24] (28.6%), while the Clinical Evaluation of Language Fundamentals (CELF) was similarly employed in two investigations [23,25] (28.6%). These instruments emerged as the most frequently applied measures across the reviewed literature, reflecting an equivalent distribution between the PLS and CELF protocols in the analyzed research. In contrast, neuropsychological assessments exhibited substantial variability across the studies, with no single standardized instrument predominating.

#### 3.4.2. Auditory Assessment

Concerning the peripheral auditory profile, three studies did not specify the audiological examinations performed in their methodology; however, they explicitly stated that the target population presented hearing thresholds within normal clinical limits [21,23,25]. Among the four studies that reported specific protocols, a combination of pure-tone audiometry, speech audiometry, and tympanometry was utilized in two investigations (50.0%) [20,24]. One study restricted its peripheral assessment to pure-tone audiometry combined with tympanometry [22] (25.0%), whereas another relied exclusively on tympanometry to verify middle ear status [26] (25.0%). Finally, among the four studies that detailed their audiological procedures, only two provided specifications regarding the equipment used to perform the examinations.

### 3.5. Auditory Electrophysiological Assessments

Auditory Brainstem Response (ABR) testing [20,24,25,26] (57.1%) and the Frequency-Following Response (FFR) [20,24,25] (42.9%) emerged as the most frequently utilized electrophysiological measures among the reviewed literature. These were followed by long-latency auditory evoked potentials (LLAEPs) [22,24] (28.6%). Conversely, auditory steady-state responses (ASSR) and the N400 potential were each investigated in only one study, representing 14.3% of the sample [21,26]. The specific procedures, equipment, parameters, and findings for each auditory evoked potential are detailed below. Table 3 presents a direct comparison of the potential assessed, the direction of the group difference, and the corresponding GRADE certainty rating for each of the seven included studies and may be consulted alongside the narrative synthesis below for an at-a-glance comparison of effect direction across studies.

#### 3.5.1. Auditory Brainstem Response (ABR)

*Procedures and Equipment*: ABR testing was implemented in four of the seven included studies (57.1%): Gabr et al. [20], Elmahallawi et al. [24], Barman et al. [25], and Aras et al. [26]. Among these, only two investigations specified the commercial systems employed. The Smart-EP™ (Intelligent Hearing Systems—IHS, Miami, FL, USA) was utilized in two studies (28.6%) [20,24], whereas the Eclipse EP25™ (Interacoustics, Middelfart, Denmark) [26] and the Bio-logic^®^ Navigator^®^ Pro (Natus Medical, Mundelein, IL, USA) [25] were each employed in a single study (14.3%).

*Technical Parameters*: Only two of the four studies that utilized ABR provided a description of the recording and stimulation parameters (representing 28.6% of the total included studies). Gabr et al. [20] reported a stimulus intensity of 90 dB nHL and a recording protocol consisting of 3 sweeps of 1024 repetitions within an analysis window of 0–75 ms; however, other critical parameters (such as stimulus duration, presentation rate, high-pass and low-pass filter settings, gain, artifact rejection, polarity, and electrode montage) were not reported (NR). Conversely, Aras et al. [26] specified a stimulus duration of 10 ms, an intensity of 70 dB nHL, a presentation rate varying between 27 and 65 Hz, a recording protocol of 1 sweep of 2000 repetitions, and an analysis window of 0–75 ms.

*Findings across Studies*: Across the four studies that assessed click-ABR, the direction of findings was consistent in three investigations, which reported no significant group difference under conventional fixed-rate stimulation, and divergent in one investigation, which identified a significant difference only after the stimulus presentation rate was experimentally manipulated. In the investigation by Gabr et al. [20], the core ABR waveforms were identifiable in 100% of the participants across both the control and DLD groups, with no statistically significant differences observed between the cohorts. Consistent with these findings, Elmahallawi et al. [24] reported that wave latencies and amplitudes did not demonstrate significant discrepancies between children with DLD and typically developing controls. Notably, in both investigations, the conventional click-ABR was utilized primarily as a complementary screening procedure to ensure brainstem integrity prior to the FFR, which served as the primary objective of their research. Furthermore, while Barman et al. [25] conducted ABR testing as a preliminary assessment to verify peripheral and lower-brainstem pathway normalcy, the specific quantitative results for this potential were not explicitly reported in their paper; this outcome was therefore treated as missing data for this study and excluded from the directional comparison above, rather than being counted as a finding of no difference. Finally, Aras et al. [26] was the only included study to explore ABR as a primary outcome measure. Their findings indicated that children with DLD exhibited prolonged I–III and I–V interpeak intervals compared to the reference database. Additionally, longer wave latencies were observed as the stimulus presentation rate increased, whereas a progressive reduction in latencies was noted as a function of the participants’ advancing age.

#### 3.5.2. Auditory Steady-State Responses (ASSR)

*Procedures and Equipment*: Of the seven articles selected for this review, one [26] (14.3%) reported the use of ASSR within its methodology. This study incorporated the ASSR as a complementary screening measure, utilizing the Eclipse EP25™ system (Interacoustics, Middelfart, Denmark) (14.3%).

*Technical Parameters*: The study did not provide a detailed description of the acquisition or stimulation parameters utilized for the ASSR recordings.

*Findings across Studies*: According to the authors, the ASSR served as a complementary measure to verify the peripheral auditory status of the children with DLD enrolled in the study, all of whom were classified as having normal hearing, with thresholds remaining within 20 dB HL. As ASSR was reported in a single study, no cross-study comparison of effect direction was possible for this outcome; the finding is therefore reported descriptively rather than synthesized.

#### 3.5.3. Frequency-Following Response (FFR)

*Procedures and Equipment*: Regarding the FFR, three of the seven articles included in this review (42.9%) provided information on its application within their methodology. All three studies (100% of this subset) described the equipment utilized: the Smart-EP™ system (IHS, Miami, FL, USA) [20,24] was employed in two investigations (66.7%), while the Bio-logic^®^ Navigator^®^ Pro (Natus Medical, Mundelein, IL, USA) [25] was used in one study (33.3%).

*Technical Parameters*: Methodological variability was observed across the studies regarding stimulus duration, filter settings, analysis windows, and intensities. In all three analyzed studies [20,24,25], stimulus duration parameters were reported, ranging between 40 ms and 206 ms, with the speech syllable/da/being consistently employed as the acoustic stimulus. Furthermore, the filter configurations and analysis windows varied: one study utilized a 50 Hz high-pass filter and a 1000 Hz low-pass filter with an analysis window of 0–12 ms [20]; another employed a 100–3000 Hz band-pass filter with a 75 ms analysis window [24]; and the third used a 30–1500 Hz band-pass filter with a 100 ms observation window [25].

Regarding stimulus intensity, two studies (66.7%) utilized 80 dB, specified in dB SPL in one investigation [25] and in dB nHL in another [24], whereas one study (33.3%) employed an intensity of 70 dB nHL [20].

In terms of presentation rates, two articles (66.7%) adopted 11.1 stimuli per second [20,24], while one (33.3%) utilized 7.1 stimuli per second [25]. Regarding amplifier gain, two studies (66.7%) applied a 100,000× gain [20,24], and one (33.3%) used a 75,000× gain [25]. Artifact rejection thresholds were specified at 31 μV in two papers (66.7%) [24,25] and at 35 μV in one (33.3%) [20]. The number of sweeps also varied, with 1024 sweeps recorded in two studies (66.7%) [20,24] and 3000 sweeps in one study (33.3%) [25].

Finally, concerning electrode montage, two articles (66.7%) utilized Fz (active), Fpz (ground), and the right and left mastoids [20,24], whereas one study (33.3%) employed a montage consisting of Fz (active) and the right and left mastoids [25].

*Findings across Studies*: All three studies reported the direction of FFR alterations between DLD and control groups using comparable latency and amplitude metrics, allowing comparison of effect direction and breadth across studies despite differences in the specific wave components analyzed. In their investigation, Gabr et al. [20] observed that individuals diagnosed with DLD tended to exhibit prolonged latencies and reduced amplitudes for waves A, C, D, E, F, and O compared to the control group. Furthermore, regarding the V-A complex, the authors reported a reduction in both the amplitude and duration of this component in children with DLD relative to their typically developing peers.

Conversely, Elmahallawi et al. [24] highlighted distinct outcomes when investigating the FFR in children with DLD compared to those with typical language development. The authors analyzed performance across three testing conditions: in quiet and under two signal-to-noise ratios (+5 dB and +10 dB). According to their report, prominent discrepancies between the groups were not observed in quiet conditions. However, in background noise conditions, both groups demonstrated a degradation in performance, with children with DLD exhibiting more pronounced vulnerability, as evidenced by sharper alterations in their results.

Barman et al. [25] demonstrated that children with DLD exhibited significantly prolonged latencies in waves C and D bilaterally when compared to the typically developing group, potentially reflecting a delay in the subcortical processing of information related to speech stimulus transitions at the brainstem level. In contrast, statistically significant differences were not observed in the latencies of waves V, A, E, F, and O, nor in amplitude measures, which may suggest that the magnitude of the neural response was not the primary altered factor. Additionally, variations in response morphology were identified; while typically developing children displayed well-defined waveforms with sharper peaks and higher neural synchrony, the children with DLD presented less organized responses, characterized by reduced peaks and lower brainstem neural synchronization. It should be noted that the study relied on a limited sample size of 10 participants, an aspect that limits the reliability of the findings and increases susceptibility to bias during data analysis. Taken together, the latency of FFR affected components varied considerably across studies, ranging from most waveform components [20] to alterations restricted to two components [25], while Elmahallawi et al. [24] found no difference under quiet conditions but pronounced impairment under noise indicating that protocol-specific listening conditions and stimulus parameters may modulate the apparent sensitivity of the FFR to DLD-related alterations.

#### 3.5.4. Long-Latency Auditory Evoked Potentials (LLAEP: Cortical Potentials and P300)

*Procedures and Equipment*: Regarding long-latency auditory evoked potentials, one of the seven included studies (14.3%) incorporated cortical auditory evoked potentials into its methodology [23], while another investigation (14.3%) evaluated both cortical and cognitive (P300) potentials [22]. Concerning the instrumentation utilized, only one article (14.3%) specified the commercial system employed, which consisted of the LabChart™ data acquisition system (ADInstruments Ltd., Dunedin, New Zeland) [22].

*Technical Parameters*: Only one article [22] (14.3%) detailed the stimulation and recording parameters, reporting the use of a conventional auditory oddball paradigm. This protocol featured a 500 Hz tone as the standard stimulus (80% probability) and a 2 kHz tone as the deviant/rare stimulus (20% probability); both presented bilaterally.

*Findings across Studies*: The investigation by Wlodarczyk et al. [22] evaluated children aged 7 to 10 years. Their findings indicated that LLAEP latencies tended to vary as a function of age, with a progressive reduction in the latencies of both the N2 and P300 components observed alongside advancing chronological age. However, children diagnosed with DLD exhibited significantly prolonged latencies, particularly regarding the P300 component, when compared to the control group across all evaluated age groups. Additionally, children with DLD aged 8 to 10 years demonstrated a significant increase in N2 latency, whereas the 7-year-old subgroup exhibited a similar descriptive trend that did not reach statistical significance.

In the study by Kwok et al. [23], the findings suggested that the maturation of cortical auditory responses accounted for approximately 31% of the variance in the linguistic abilities of the evaluated children, highlighting a potential link between central auditory processing development and language performance. Notably, children with mild DLD exhibited auditory responses comparable to those of the control group, whereas individuals presenting moderate-to-severe DLD demonstrated a delay in the maturation of cortical auditory responses, estimated at approximately 1.3 years. Although [22,23] examined cortical maturation through different analytical lenses, chronological age and DLD severity, respectively, both studies converge in identifying a maturational lag in children with DLD relative to typically developing peers, suggesting that this delay may be a consistent feature of LLAEP findings across studies despite differing analytical approaches.

#### 3.5.5. N400 Potential

*Procedures and Equipment*: Within the domain of LLAEPs, the N400 potential was also explored. Among the seven selected articles, a single investigation (14.3%) incorporated the N400 into its methodology [21]; however, a description of the equipment utilized for the examination was not provided by the authors.

*Technical Parameters*: The study did not report on the specific acquisition or stimulation parameters used for the N400 recordings.

*Findings across Studies*: The findings reported by Pijnacker et al. [21] revealed discrepancies in semantic processing between children with DLD and typically developing peers. The typically developing group displayed a well-defined N400 effect, characterized by greater response amplitudes to semantically incongruent words compared to congruent ones within the 300–500 ms time window. In contrast, the group with DLD did not exhibit this effect during the initial time window; instead, they demonstrated a delayed and atypical response pattern, with the N400 effect becoming apparent only within the subsequent 500–800 ms window. As the N400 was reported in a single study, no cross-study comparison of effect direction was possible for this outcome; the finding is therefore reported descriptively rather than synthesized.

### 3.6. Evidence Certainty Synthesis (GRADE)

To estimate the certainty of the body of evidence for each electrophysiological outcome, the GRADE approach was applied across included studies. Confidence levels ranged from very low (N400, ASSR) to moderate (LLAEP/P300), reflecting the combined impact of risk of bias, methodological inconsistency, and sample imprecision (Table 8). These classifications should be considered when interpreting the clinical relevance of the findings described in the following sections.

## 4. Discussion

The present study aimed to systematically review the available evidence regarding the relationship between auditory evoked potentials (AEPs) and Developmental Language Disorder (DLD). To facilitate clarity and ensure a logical flow for the reader, the discussion is structured according to the primary thematic categories outlined in the results section.

### 4.1. Sample Characteristics

Regarding sample characteristics, the concentration of participants below 10 years of age highlights a noticeable gap in the literature concerning older children and adolescents. It is worth noting that while the present review intended to evaluate evidence for individuals with DLD up to 12 years of age, none of the retrieved studies included participants over the age of 10. This absence appears to be clinically relevant, given that DLD is recognized as a persistent disorder extending into adolescence and adulthood, with documented impacts on academic achievement and quality of life. Consequently, the scarcity of electrophysiological studies within this older cohort substantially limits the understanding of the neurobiological trajectory of the disorder across development [9,27,28]. Supporting these observations, previous research has indicated that language difficulties identified during the preschool years often persist, highlighting the importance of monitoring these children into adolescence rather than if such impairments will resolve spontaneously with age [10].

Concerning sex distribution, only three of the seven included studies explicitly reported this variable, which itself represents a significant limitation in sample characterization. Based on the available data from these studies, an asymmetrical pattern between groups emerged: while the control groups exhibited a predominance of female participants, the DLD cohorts were composed primarily of males. This distribution aligns with epidemiological literature indicating a higher probability of DLD diagnoses in boys than in girls [3,29,30]. Current literature notes that the reported male-to-female ratio may vary depending on the specific language profile, with estimates for DLD reaching approximately 3:1 [31]. However, evidence suggests a potential sex bias in identification. Boys may be more likely to be identified and receive both parental [32] and clinical [33] attention for language-related challenges, rather than this asymmetry purely reflecting an inherent biological susceptibility to DLD—a phenomenon frequently termed referral bias.

Therefore, the findings of the present review should be interpreted with caution, as it remains challenging to determine whether the observed differences in auditory evoked potentials reflect a generalized pattern of DLD or are partially modulated by the specific sex composition of the studied samples. Furthermore, the omission of sex-related information in several studies precludes stratified analyses and restricts the systematic exploration of potential interactions among biological sex, auditory maturation, and electrophysiological parameters [34,35]. This aspect remains critical, as neurobiological and hormonal factors may modulate the maturation of auditory pathways as well as performance on linguistic tasks [36,37].

### 4.2. Language Assessment

The application of the Preschool Language Scales (PLS) [20,24] and the Clinical Evaluation of Language Fundamentals (CELF) [23,25] in equal proportions across the analyzed studies may reflect the heterogeneity of the investigated samples. Although both instruments are widely validated, they feature distinct clinical structures: the PLS is typically directed toward preschoolers, focusing primarily on auditory comprehension and expressive communication, whereas the CELF spans broader age brackets and evaluates multiple linguistic domains via subtests for phonology, semantics, syntax, and working memory [38]. Furthermore, investigations such as Aras et al. [26] utilized instruments that are not widely adopted internationally (e.g., the Croatian version of the Reynell Developmental Language Scales III), which potentially complicates the cross-cultural comparability of the findings. Similarly, Pijnacker et al. [21] employed a combination of the PPVT-Dutch, STvT, and STT-II, configuring a battery tailored to the Dutch context but contributing to the overall methodological heterogeneity among the reviewed studies.

Another relevant consideration is the absence of standardized criteria to define the severity of DLD, which represents critical information for interpreting the magnitude of electrophysiological findings. Only Kwok et al. [23] stratified participants by severity (mild vs. moderate-to-severe), demonstrating that this variable substantially modulates cortical responses. Such heterogeneity in diagnostic criteria and the lack of severity stratification has been identified by the CATALISE-2 consensus [2] as persistent obstacles to cross-study comparisons in DLD research. Compounding this issue, the wide variability of the neuropsychological instruments used suggests the absence of a standardized assessment protocol. Given that the CATALISE consensus [2] eliminated the requirement for a discrepancy between nonverbal IQ and linguistic performance for a DLD diagnosis while maintaining the recommendation to exclude intellectual disability, the omission of cognitive measures in certain studies may represent a methodological limitation that could potentially compromise sample specificity.

### 4.3. Auditory Assessment

Analysis of the audiological protocols employed across the included studies revealed substantial heterogeneity and partially incomplete methodological reporting. Three of the seven studies did not specify the audiological tests performed, limiting their description to stating that participants exhibited hearing thresholds within normal limits [20,23,24]. Among the four studies that reported this information, only Gabr et al. [18] and Elmahallawi et al. [23] adopted a comprehensive protocol comprising pure-tone audiometry, speech audiometry, and immittance testing—a combination internationally recognized as the minimum standard for characterizing peripheral auditory status and middle ear function [39]. Conversely, Wlodarczyk et al. [22] utilized pure-tone audiometry and tympanometry without speech audiometry, while Aras et al. [26] relied exclusively on tympanometry, an approach that, in isolation, does not allow for the quantification of air- and bone-conduction hearing thresholds.

The relevance of this heterogeneity lies in the pivotal role that auditory evaluation occupies within the diagnostic process of DLD. The CATALISE-2 consensus [2] establishes the exclusion of hearing loss as a fundamental prerequisite for a DLD diagnosis to ensure that language impairments are not primarily attributable to peripheral deficits, particularly because even mild or fluctuating conductive hearing loss can induce delays in language development. Each component of the basic audiological battery addresses a distinct mechanism: pure-tone audiometry quantifies air- and bone-conduction thresholds, allowing for the detection of sensorineural hearing loss and its differentiation from conductive pathologies; speech audiometry assesses the recognition of speech stimuli and the integration between acoustic perception and foundational linguistic processing; and immittance testing evaluates middle ear integrity and stapedial reflex thresholds, showing high sensitivity for detecting Eustachian tube dysfunction and otitis media with effusion (OME). Therefore, a complete basic audiological evaluation appears essential to preclude the inclusion of children with undetected peripheral auditory deficits, which could otherwise confound the interpretation of electrophysiological responses. It should be noted, however, that conventional speech audiometry probes word recognition monaurally (one ear at a time) and therefore does not assess binaural integration. The integration of the differing signals reaching the two ears is computed subcortically at the superior olivary complex and is more directly probed by dichotic listening paradigms, none of which were employed by the included studies.

### 4.4. Auditory Electrophysiological Assessments

*Auditory Brainstem Response (ABR)*: ABR testing was the most frequently reported electrophysiological procedure among the studies, employed to verify the structural integrity of the auditory pathway up to the brainstem [20,24,25]. Based on the results from Gabr et al. [20] and Elmahallawi et al. [24], no statistically significant differences between the groups (DLD and typical development) were identified, which may suggest that the conventional click-ABR might lack the sensitivity required to detect subtle anomalies in individuals with primary language impairments [27,40]. Conversely, the implementation of click-ABR protocols involving modifications to stimulus presentation parameters, as proposed by Aras et al. [26], identified alterations in interpeak intervals. These results could be interpreted considering hypotheses reporting an impairment in processing rapid acoustic transitions, which could demand higher neural synchronization [13,14]. Consequently, the findings of this review suggest that the traditional click-ABR may possess limited diagnostic utility in DLD when used in isolation but could potentially gain sensitivity when applied within temporal stress paradigms. Thus, manipulating presentation rates represents a promising avenue for exploration in future investigations.

*Auditory Steady-State Response (ASSR)*: ASSR was mentioned in only one study [26], in which it was employed as a complementary tool to confirm participants’ hearing thresholds rather than as a primary variable of interest for comparative analysis between groups.

*Frequency-Following Response (FFR)*: The FFR occupied a prominent position among the analyzed studies, serving as the primary procedure in three of the included investigations [20,24,25]. As detailed in Section 3.5.3, the consistency of these findings varied across studies, ranging from near-global alterations to changes restricted to specific components, with noise-degraded conditions further modulating the apparent sensitivity of the response. One potential hypothesis for this pattern of variation is that it might be attributed to differences in stimulus duration across the protocols, since longer stimuli tend to favor the sustained, periodicity-related portion of the response, whereas shorter stimuli emphasize its transient onset components; consequently, studies employing different stimulus durations may be differentially sensitive to distinct neural generators along the same subcortical pathway, which would account for the partially divergent component-level findings without necessarily implying inconsistent underlying pathophysiology.

These results suggest that individuals with DLD may exhibit alterations in the neural encoding of speech stimuli, which could potentially compromise the representation of acoustic speech cues and impact perception, especially under challenging listening conditions [41]. Additionally, as reported by Elmahallawi et al. [24], while a degradation in performance under noise was verified in both cohorts, children with DLD showed a higher degree of impairment, evidenced by more pronounced alterations in their responses. Therefore, this assessment could represent a valuable tool for exploring neurophysiological mechanisms in individuals with DLD and evaluating the consequences of impaired speech-sound encoding. At the subcortical level, the temporal precision indexed by the FFR depends on sub-millisecond neural timing within the brainstem, including the binaural circuitry of the superior olivary complex and specialized synapses such as the calyx of Held. Atypical encoding at this level could plausibly contribute to the FFR alterations observed in DLD, a mechanism not directly tested by the reviewed protocols.

*Long-Latency Auditory Evoked Potentials (LLAEP) and P300*: Long-latency auditory evoked potentials to evaluate cortical auditory processing and reflect more elaborate stages of stimulus analysis involving memory, attention, and discrimination. The exogenous components (P1, N1, P2, and N2) are modulated by the physical characteristics of the stimulus and the structural maturation of the auditory pathway, whereas the endogenous P300 component is thought to reflect cognitive processing, resource allocation, memory updating, and decision-making [42].

Wlodarczyk et al. [22] and Kwok et al. [23], the two reviewed studies that implemented LLAEPs, converged on several key findings. First, both documented a reduction in cortical component latencies with advancing age, a pattern consistent with the expected neurobiological maturation of the auditory cortex. Second, both identified that children with DLD tended to exhibit prolonged latencies compared to typically developing peers, particularly within the N2 and P300 components. Wlodarczyk et al. [22] observed that the prolongation of the N2 wave was more evident between 8 and 10 years of age, a developmental window in which cortical maturation typically supports highly synchronized neural responses. Kwok et al. [23] demonstrated that the maturation of cortical potentials accounted for approximately 31% of the variance in linguistic abilities and estimated an average delay of 1.3 years in the cortical maturation of children with moderate-to-severe DLD.

These insights are valuable as they suggest that anomalies in these procedures might predict more than the mere presence or absence of an impairment in DLD; rather, the magnitude of the alteration may reflect the severity of linguistic compromise. This interpretation aligns with the procedural deficit hypothesis proposed by Ullman and Pierpont [43], which posits that DLD may stem from dysfunctions within brain circuits responsible for implicit procedural learning—systems heavily reliant on the maturational integration between subcortical structures (basal ganglia) and cortical regions (frontal and parietal cortices).

The P300 component, specifically, appeared sensitive to DLD in the findings of Wlodarczyk et al. [22], demonstrating prolonged latencies across all evaluated age groups. This component reflects the allocation of attentional resources toward relevant auditory stimuli within oddball paradigms and is linked to auditory working memory and discrimination. Prolonged P300 latencies in DLD suggest that a greater duration may be required to discriminate and categorize acoustic signals, which clinically translate into challenges tracking speech under conditions demanding heightened attentional support—such as classroom environments, group conversations, or multi-talker settings. A meta-analysis by Silva et al. [44] corroborated this trend, indicating that individuals with language disorders exhibit systematically prolonged P300 latencies relative to unaffected peers, and that speech-language intervention can modulate these parameters, offering valuable evidence of neural plasticity relevant to therapeutic planning.

A systematic review by Hosseini-Foladkar et al. [45] focusing on cortical auditory evoked potentials (CAEPs) in learning disabilities strengthens the interpretation that cortical measures represent promising markers for differentiating neurodevelopmental disorders, although the clinical overlap among DLD, dyslexia, and ADHD may limit diagnostic specificity. For illustrative purposes, a case report by Soares et al. [27]—not included in the present review as a primary study—describing a bilateral asymmetry characterized by an absent right-hemisphere P300 and a prolonged left-hemisphere latency in an adolescent with co-occurring DLD and Specific Learning Disorder exemplifies this diagnostic overlap and reinforces the need for future studies with samples stratified by comorbidity. Taken together, the findings of the present review suggest that LLAEPs and the P300 might serve as useful indices for identifying cortical immaturity in children and adolescents with DLD. However, it is worth noting that the dichotic and binaural-integration profile of this participant was not reported; therefore, it remains difficult to confidently attribute the interhemispheric P300 to a comorbidity. Atypical hemispheric lateralization of language processing, as documented in DLD with co-occurring specific learning disorders, represents a plausible alternative explanation. This gap further underscores our broader recommendation to incorporate dichotic measures in future electrophysiological designs.

*N400 Potential*: Within the LLAEP domain, the N400 component is generally understood as an endogenous response primarily linked to semantic processing. It appears to be modulated by the coherence or incongruence between a stimulus and its linguistic context, with its amplitude tending to increase in response to semantically anomalous words within a 300–500 ms post-stimulus time window [42]. While this component is frequently regarded as a reliable marker of the temporal course of semantic integration—holding potential relevance for the study of atypical language development—some caution is warranted regarding the specific finding discussed below. Although the broader N400 literature is well-established, the particular result reported here stems from a single study with a very low GRADE rating, suggesting that it should be interpreted conservatively.

The only investigation within this review to evaluate the N400 was conducted by Pijnacker et al. [21], who reported an atypical response pattern in preschoolers with DLD, potentially suggesting a form of temporal inefficiency that may necessitate an extended duration to integrate semantic information into a given context. Research by Lindfors et al. [46] highlighted that N400 alterations in DLD might not be restricted solely to the verbal domain but could also extend to pictorial tasks. This suggests the involvement of broader cognitive networks, reinforcing the hypothesis that DLD may not be an exclusively linguistic disorder, a view consistent with the assertions of Ullman and Pierpont [43]. Given that these findings rely on a single study, however, the extent to which broader inferences can be drawn remains constrained, underscoring the importance of additional research to better clarify how the N400 potential relates to DLD profiles. In this context, the observed temporal inefficiency refers specifically to a protracted course of cortical semantic integration, reflected by the delayed 500–800 ms N400 effect, rather than reflecting a brainstem-level timing deficit.

### 4.5. Risk of Bias Assessment

The risk of bias assessment using the Joanna Briggs Institute (JBI) criteria indicated a moderate methodological quality among the seven studies included in this review. The electrophysiological procedures employed and the statistical analysis of the data were considered adequate. Conversely, limitations were identified in the identification and control of potential confounding factors, suggesting that relevant variables—such as neurodevelopmental comorbidities, socioeconomic status, cognitive profiles, and intervention history—were not systematically accounted for. This omission may limit the robustness of the inferences regarding the specific association between DLD and the observed alterations in evoked potentials. Additionally, the included studies exhibited substantial methodological heterogeneity, underscoring the need for future investigations with enhanced scientific rigor, particularly concerning sample design and the control of confounding variables, to ensure that the association between DLD and auditory electrophysiological measures can be accurately estimated and effectively contribute to the assessment and monitoring of this population.

### 4.6. Evidence Certainty Synthesis (GRADE)

The GRADE approach enabled an individual analysis of each electrophysiological procedure, revealing that the certainty of evidence was classified as low for both the FFR and click-ABR, moderate for the N2 and P300 components, and very low for the N400 potential and ASSR. Consistent with the JBI appraisal, inconsistencies across study findings and reduced sample sizes were observed. These classifications reflect a combination of serious risk of bias in a substantial portion of the studies, methodological heterogeneity (encompassing stimulus protocols, age ranges, and DLD diagnostic criteria), and imprecision due to small sample sizes, which collectively limits the degree of confidence regarding the electrophysiological responses in children with DLD compared to their typically developing peers.

#### Sources of Heterogeneity and Their Impact on the Consistency of Findings

The heterogeneity underlying these certainty ratings appears to stem from at least three distinct, yet partially overlapping, sources. The first concerns stimulus and protocol heterogeneity: included studies varied considerably in stimulus type, duration, and presentation rate—factors that may differentially affect the transient and sustained components of the auditory response. This is illustrated by the divergent FFR findings between Gabr et al. [18], who reported prolonged latencies and reduced amplitudes across nearly all components, and Barman et al. [24], who observed alterations restricted to waves C and D. This discrepancy might be partially attributed to differences in stimulus duration and presentation parameters between the two protocols (see Section 4.4 for further discussion). Similarly, click-ABR findings diverged between studies using conventional fixed-rate paradigms [18,23], which did not detect group differences, and Aras et al. [25], who identified prolonged interpeak intervals only after manipulating the stimulus presentation rate. This suggests that protocol design itself might influence whether a given potential appears sensitive to DLD.

The second source relates to sample heterogeneity, encompassing variations in age range, DLD severity, and comorbidity profiles across studies. The LLAEP findings illustrate this directly, as Kwok et al. [22] noted that the magnitude of cortical maturation delay differed substantially between children with mild DLD—who showed responses comparable to typically developing peers—and those with moderate-to-severe DLD, who exhibited an average 1.3-year maturational delay. The third source involves outcome-definition and acquisition of heterogeneity, including differing electrode montages, filter settings, and criteria for component identification. As detailed in Section 3.5.4, the included LLAEP studies employed non-identical electrode arrangements, which could partially account for between-study variability in the reported latencies for the N2 and P300 components. Collectively, these three sources of heterogeneity do not merely preclude statistical pooling; they likely contribute to the apparent inconsistency of findings within the same electrophysiological potential. Consequently, this indicates that the comparative interpretation of evoked potentials across the literature should ideally account for protocol-level and sample-level variation, rather than attributing inconsistent findings solely to the type of potential.

### 4.7. Limitations

The present review is subject to certain methodological limitations that warrant consideration when interpreting the findings. First, while the ten-year time frame (January 2016–March 2026), was appropriate for capturing post-CATALISE nomenclature, it might have inadvertently excluded older studies conducted under SLI criteria that could potentially have provided complementary evidence. Second, the relatively number of included studies (*n* = 7) and the substantial heterogeneity in stimulus protocols, DLD diagnostic criteria, sample ages, and outcome reporting precluded meta-analytic pooling and tended to constrain the generalizability of the narrative synthesis. Third, only six of the seven included studies featured a typically developing control group; the absence of such a cohort in one investigation (Aras et al.) appears to limit cross-study comparability for specific outcomes. Finally, a formal assessment of publication bias was not feasible given the limited number of studies per outcome, and the possibility that studies with null or non-significant findings remain unpublished cannot be entirely ruled out.

A further, cross-cutting limitation of the reviewed evidence is that auditory function was assessed monaurally (one ear at a time) in every included study, both behaviorally and electrophysiologically. Because all auditory input is ultimately integrated at the superior olivary complex—the subcortical gateway for auditory scene analysis, selective auditory attention, dichotic listening, and speech perception—measures restricted to a single ear may not fully characterize binaural integration. Future studies might therefore benefit from (i) complement ear-specific acuity with dichotic listening tasks in both groups, and (ii) recording the auditory potentials from the right and left ears separately, as well as under binaural stimulation, to explicitly evaluate interaural differences. Framing the limitations of this literature solely in terms of participant age and protocol heterogeneity would likely understate the significance of this gap.

### 4.8. Clinical Implications for Audiology and Speech-Language Pathology

The present findings may offer practical guidance for selecting auditory evoked potentials according to clinical purposes. Conventional click-ABR appears primarily suited as a screening tool to exclude peripheral and lower-brainstem pathology prior to further testing; given its limited standalone sensitivity to isolated DLD, it might be less appropriate as a differential marker. The FFR, by contrast, demonstrated the greatest sensitivity to subcortical speech-encoding deficits, particularly under degraded listening conditions. This suggests that it could hold greater value for differential characterization of children with suspected DLD, especially when behavioral auditory processing tests are constrained by attention or cooperation. Furthermore, the P300 and other LLAEPs—which index cortical maturation, attentional allocation, and discrimination—appear relevant not only for diagnostic characterization but also for longitudinal monitoring of intervention outcomes. For instance, the meta-analysis by Silva et al. [44] indicated that speech-language therapy was associated with reducing P300 latencies in individuals with language disorders. This suggests that this potential might be sensitive to therapy-induced neural plasticity, potentially serving as an objective, quantifiable outcome measure to track treatment response over time alongside standardized language assessments.

Taken together, these findings seem to support the consideration of a tiered clinical application: ABR for peripheral/brainstem screening, FFR for differential diagnostic characterization of subcortical encoding deficits, and P300/LLAEP for both diagnostic support and intervention monitoring. Importantly, none of these measures would be advisable as a stand-alone diagnostic criterion for DLD; rather, they are perhaps best viewed as objective adjuncts within a comprehensive, multidisciplinary audiological and speech-language evaluation protocol.

## 5. Conclusions

This systematic review suggests that children with DLD may exhibit a pattern of auditory electrophysiological alterations across subcortical and cortical levels. Among the investigated potentials, the FFR and LLAEP/P300 appear to be relatively more sensitive measures for this population. However, it is important to note that the specific latency and amplitude of the observed FFR alterations varied considerably across studies. Furthermore, as the certain of evidence remains low for the FFR and moderate for the LLAEP/P300, these findings should be interpreted with caution. Regarding other measures, click-ABR results remain inconsistent across literature; evidence concerning the N400 is currently limited to a single investigation; and the ASSR has been predominantly utilized to rule out peripheral hearing loss rather than to characterize DLD. From a clinical perspective, FFR and LLAEP/P300 assessments might serve as objective neurophysiological adjuncts to behavioral language evaluation, particularly when standardized testing is limited by age or patient cooperation. To advance the field, future research would benefit from standardizing electrophysiological protocols, extending investigations to adolescents and young adults, and employing longitudinal designs to further explore the predictive validity of these measures for language outcomes.

## Figures and Tables

**Table 1:** Guiding research questions formulated based on the PICO framework and its constituent elements.

Population (P)	Children and Adolescents (Aged 1–12 years) with a Confirmed Clinical Diagnosis of Developmental Language Disorder (DLD) and Peripheral Hearing Thresholds Within Normal Limits.
Intervention (I)	Assessment utilizing auditory evoked potentials (AEPs).
Comparison (C)	Typically developing children and adolescents, whether matched or unmatched for age and sex
Outcome (O)	Discrepancies in latency, amplitude, morphology, or the presence/absence of auditory evoked potential waveforms between the study and comparison groups.

**Table 2:** Database search strategy using descriptors combined with Boolean operators.

Database	Combination Search Terms	Total Results
PubMed	(“Developmental Language Disorder” OR “DLD” OR “Specific Language Impairment” OR “SLI” OR “Language Disorders” OR “Language Impairment”) AND (“Evoked Potentials, Auditory” OR “Auditory Evoked Potentials” OR “Auditory Brainstem Response” OR “ABR” OR “BAEP” OR “Frequency-Following Response” OR “FFR” OR “Event-Related Potentials” OR “P300” OR “LLAEP” OR “Late Auditory Evoked Potentials” OR “Cortical Auditory Evoked Potentials”)	97
Web of Science	TS = (“Developmental Language Disorder” OR “DLD” OR “Specific Language Impairment” OR “SLI” OR “Language Disorders” OR “Language Impairment”) AND TS = (“Evoked Potentials, Auditory” OR “Auditory Evoked Potentials” OR “Auditory Brainstem Response” OR “ABR” OR “BAEP” OR “Frequency-Following Response” OR “FFR” OR “Event-Related Potentials” OR “P300” OR “LLAEP” OR “Late Auditory Evoked Potentials” OR “Cortical Auditory Evoked Potentials”)	154
Scopus	TITLE-ABS-KEY(“Developmental Language Disorder” OR “DLD” OR “Specific Language Impairment” OR “SLI” OR “Language Disorders” OR “Language Impairment”) AND TITLE-ABS-KEY(“Evoked Potentials, Auditory” OR “Auditory Evoked Potentials” OR “Auditory Brainstem Response” OR “ABR” OR “BAEP” OR “Frequency-Following Response” OR “FFR” OR “Event-Related Potentials” OR “P300” OR “LLAEP” OR “Late Auditory Evoked Potentials” OR “Cortical Auditory Evoked Potentials”)	198
	Total number of articles	449

**Table 3:** Key findings by study, electrophysiological potential, findings, and GRADE certainty of evidence.

Study	Potential Assessed	Findings	GRADE Certainty
Gabr et al., 2016 [20]	ABR/FFR	ABR: No significant group differences. FFR: prolonged latencies and reduced amplitudes across nearly all components (DLD < control).	LOW (FFR)/LOW (ABR)
Pijnacker et al., 2017 [21]	N400	Absent N400 effect in the early window (300–500 ms); delayed, atypical N400 effect emerging only at 500–800 ms in DLD.	VERY LOW
Wlodarczyk et al., 2018 [22]	LLAEP (N2/P300)	Prolonged N2 and P300 latencies in DLD across all age groups, most evident at 8–10 years for N2.	MODERATE
Kwok et al., 2018 [23]	LLAEP (cortical AEPs)	Mild DLD: responses comparable to controls. Moderate-to-severe DLD: ~1.3-year delay in cortical auditory maturation	MODERATE
Elmahallawi et al., 2022 [24]	ABR/FFR	ABR: no significant group differences. FFR: no difference in quiet; greater impairment in DLD under noise (reduced F0/F2 amplitude).	LOW (FFR)/LOW (ABR)
Barman et al., 2022 [25]	ABR/FFR	ABR: results not explicitly reported. FFR: prolonged latencies in waves C and D and poorer morphology in DLD; waves V, A, E, F, O unaffected.	LOW (FFR)/LOW (ABR)
Aras et al., 2024 [26]	ABR/ASSR	ABR: prolonged I–III and I–V interpeak intervals in DLD (and other clinical subgroups) vs. reference database. ASSR used only to confirm normal hearing thresholds (not a comparative outcome).	LOW (ABR)/VERY LOW (ASSR)

Note: GRADE ratings correspond to the outcome-level certainty reported in Table 7, applied here to the specific potential each study evaluated. ABR = Auditory Brainstem Response; FFR = Frequency-Following Response; LLAEP = Long-Latency Auditory Evoked Potentials; ASSR = Auditory Steady-State Response; DLD = Developmental Language Disorder.

**Table 4:** Data extraction from the studies included in the systematic review.

Author/Year	Sample Characteristics	Electrophysiological Assessment	Complementary Assessment	Equipment	Results	Conclusion
Gabr et al., 2016 [20]	CG: 20 (3 to 7 years; mean age 5.4 ± 1.45 years); 9 males; 11 females SG: 20 (3 to 7 years; mean age 4.4 ± 1.98 years); 10 males; 10 females	ABR FFR	(PLS-4) Arabic edition Otological examination PTA Speech audiometry Tympanometry	AC40™—Interacoustic, Middelfart, Denmark. Madsen Zodiac 901™—GN Otometrics, Taastrup, Denmark Smart-EPs™—IHS, Miami, FL, USA ER3A insert phone	All ABR waves were detected in 100% of cases in both groups. Regarding FFR, children with DLD showed significantly prolonged latencies (waves A, C, D, E, F, and O) and reduced amplitudes compared to the control group. In the VA complex, children with DLD presented reduced amplitude, shorter duration, and decreased slope, with no significant difference in area between the groups	Children with DLD show alterations in the neural encoding of speech stimuli, with abnormal FFR responses, suggesting deficits in temporal auditory processing and neural synchrony
Pijnacker et al., 2017 [21]	CG: 25 (4 years and 2 months to 6 years and 5 months); NR SG: 37 (4 years and 2 months to 6 years and 5 months); NR	Event-related potentials (ERPs)—N400	PPVT—Dutch Version STvT WNV—Dutch Edition STT-II Audiological assessments not described	NR	The results showed differences in semantic processing between children with DLD and typically developing children. Typically developing children exhibited a clear N400 effect, characterized by greater brain responses to semantically incongruent words compared to congruent words. In contrast, children with DLD did not show this effect in the early time window (300–500 ms) and displayed a delayed and atypical effect in the 500–800 ms window	The study showed that preschool children with DLD exhibit atypical sentence-level semantic processing, as evidenced by N400 electrophysiological responses
Wlodarczyk et al., 2018 [22]	CG: 100 (7 to 10 years; mean age 8.6 ± SD); 41 males; 59 females SG: 100 (7 to 10 years; mean age 8.5 ± SD); 72 males; 28 females	LLAEP (cortical and cognitive auditory evoked potentials)	Phoniatric assessment PTA Tympanometry Demmel speech assessment	CHART™—ADInstruments, Ltd., Dunedin, New Zealand	Reduction in N2 latency with increasing age (significant between 7 and 10 years). Children aged 8–10 years with DLD showed longer N2 latency than controls. Age was associated with decreased P300 latency; however, children with DLD showed prolonged P300 latency	The findings indicate a reduction in the latency of long-latency auditory evoked potentials with increasing age. However, children with DLD show longer latencies, particularly in the P300 component, compared to the CG
Kwok et al., 2018 [23]	CG: 67 (7 to 10 years); NR SG: 21 (7 to 10 years); NR	LLAEP (cortical auditory evoked potentials)	WASI CELF-4 ICC Audiological assessments not described	NR	Cortical auditory evoked potentials from 21 children with DLD were compared with 67 controls. Auditory maturation explained about 31% of the variation in language skills. Children with mild DLD showed responses similar to typically developing children, while those with moderate to severe DLD showed an average delay of about 1.3 years in cortical auditory maturation	The study concluded that children with DLD, particularly in moderate to severe cases, show delayed maturation of cortical auditory responses. This delay is mainly associated with receptive language difficulties, suggesting that immaturity in central auditory processing may contribute to language comprehension deficits
Elmahallawi et al., 2022 [24]	CG: 15 (3 to 6 years; mean age 5.04 ± 0.79 years); 4 males; 11 females SG: 25 (3 to 6 years; mean age 4.77 ± 0.85 years); 19 males; 6 females	ABR FFR	PTA Speech audiometry Tympanometry Intelligence Scale (Stanford-Binet) PLS-4	GSI 61™—Grason Stadler, Eden Prairie, MN, USA ER-3A™ Smart-EPs™—IHS, Miami, FL, USA ER3A insert phone	The authors evaluated the FFR under three conditions: in quiet and in the presence of competitive noise at signal-to-noise ratios of +5 and +10. No differences were found between children with DLD and typically developing controls in the quiet condition. However, both groups showed poorer performance in noise, with greater impairment observed in children with DLD. Additionally, children with DLD showed reduced amplitude in the F0 and F2 components	The study indicates that children with DLD show difficulties in speech perception, particularly in noisy environments. The FFR proved to be an effective method for assessing speech processing at the brainstem level, revealing a reduced ability to encode the fundamental frequency of the signal
Barman et al., 2022 [25]	CG: 5 (4 to 7 years); NR SG: 5 (4 to 7 years); NR	ABR FFR	CELF—2 Audiological assessments not described	Bio-logic™—Navigator Pro, Natus Medical Inc., Mundelein, IL, USA	Children with DLD showed longer latencies in waves C and D and poorer wave morphology compared with typically developing children, suggesting deficits in neural encoding and temporal processing of speech at the brainstem level	The ABR and FFR may help identify brainstem temporal processing deficits in children with DLD, although further studies with larger samples are needed
Aras et al., 2024 [26]	SG: 123 (2 years and 11 months to 6 years and 6 months); DLD: 39 participants ASD: 16 participants Articulation pathology: 15 participants Children with underlying organic brain pathology: 15 participants Cognitive delay: 15 participants Speech-language pathology: 23 participants	ABR ASSR	Tympanometry Brunet Lezine scale Reynell Developmental Language Scales III—Croatian version	Eclipse EP 25™—Interacoustic, Miami, FL, USA	The study found longer I–III and I–V interpeak latencies in the ABR among children with speech and language disorders, indicating alterations mainly in the early brainstem auditory pathway. The greatest delays were observed in children with organic brain lesions and Autism Spectrum Disorder, followed by DLD, while children with isolated articulatory disorders showed typical values. Latencies increased with higher stimulus rates and decreased with age, suggesting maturation of the central auditory system	The study concludes that children with language comprehension difficulties and neurodevelopmental disorders are more likely to show alterations in the Auditory Brainstem Response, particularly prolonged I–III and I–V interpeak latencies, suggesting immaturity or dysfunction in the early brainstem auditory pathways

Legend: CG—control group; SG—study group; ABR—auditory brainstem responses; PLS—Preschool Language Scale; PTA—pure-tone audiometry; LLAEP—long-latency auditory evoked potentials; PPVT—Peabody Picture Vocabulary Test (Dutch version); WNV—Wechsler Nonverbal Scale of Ability; STT-II—Schlichting Test voor Taalproductie-II; STvT—Schlichting Test for Language Comprehension; ER-3A—Etymotic Research ER-3A; CELF-4—Clinical Evaluation of Language Fundamentals, 4th edition; ICC—intraclass correlation coefficient; PLS-4—modified Preschool Language Scale, 4th edition; NR—not reported; IHS—Intelligent Hearing System; ASSR—auditory steady-state response; FFR—frequency-following response; DLD—Developmental Language Disorder; ASD—Autism Spectrum Disorder.

**Table 5:** Parameters used in relation to the electrophysiological examination performed.

Gabr et al., 2016 [20]	ABR: Stim. Duration = NR; SI = 90 dB nHL; PR = NR; HPF = NR; LPF = NR; gain = NR; AR = NR; P = NR; electrodes: NR; S = 1024; AW = 0–75 ms; three blocks of 1024 artifact-free sweeps per ear FFR: Stim. Duration = 206 ms; SI = 70 dB nHL; PR = 11.1/s; HPF = 50 Hz; LPF = 1000 Hz; gain = 100 k; AR ≈ 35 mV; P = alternating; electrodes: Fz (active), Fpz (ground), left and right mastoids (reference); S = 1024; AW = 0–12 ms; three blocks of 1024 artifact-free sweeps per ear
Pijnacker et al., 2017 [21]	N400: Stim. Duration = NR; SI = NR; PR = NR; HPF = NR; LPF = NR; gain = NR; AR = NR; P = NR; electrodes: NR; S = NR; AW = NR
Wlodarczyk et al., 2018 [22]	LLAEP: Oddball—500 Hz standard (80%) and 2 kHz oddball (20%) stimuli bilateral.
Kwok et al., 2018 [23]	LLAEP: Stim. Duration = NR; SI = NR; PR = NR; HPF = NR; LPF = NR; gain = NR; AR = NR; P = NR; electrodes: NR; S = NR; AW = NR
Elmahallawi et al., 2022 [24]	ABR: Stim. Duration = NR; SI = NR; PR = NR; HPF = NR; LPF = NR; gain = NR; AR = NR; P = NR; electrodes: NR; S = NR; AW = NR FFR: Stim. Duration = 40 ms; SI = 80 dB SPL; PR = 11.1/s; BF = 100–3000; gain = 100 k; AR ≈ 31 mV; P = alternating; electrodes: Fz (active), Fpz (ground), left and right mastoids (reference); S = 1024; AW = 75 ms
Barman et al., 2022 [25]	ABR: Stim. Duration = NR; SI = NR; PR = NR; HPF = NR; LPF = NR; gain = NR; AR = NR; P = NR; electrodes: NR; S = NR; AW = NR FFR: Stim. Duration = 40 ms; SI = 80 dB SPL; PR = 7.1/s; BF = 30–1500; gain = 75 k; AR ≈ 31 mV; P = alternating; electrodes: Fz (active), left and right mastoids (reference); S = 3000; AW = 100 ms
Aras et al., 2024 [26]	ASSR: Stim. Duration = NR; SI = NR; PR = NR; HPF = NR; LPF = NR; gain = NR; AR = NR; P = NR; electrodes: NR; S = NR; AW = NR ABR: SD = 10 ms; SI = 70 dB nHL; PR = 27–65/s; HPF = NR; LPF = NR; gain = NR; AR = NR; P = NR; electrodes: NR; S = 2000; AW = 0–75 ms

Legend: Stim. Duration—Stimulus Duration; SI—Stimulus Intensity; PR—Presentation Rate; HPF—High-Pass Filter; LPF—Low-Pass Filter; NR—Not Reported; AR—Artifact Rejection; P—Polarity; S—Sweeps; AW—Analysis Window; BF—Bandpass Filter.

**Table 6:** Methodological quality assessment of analytical cross-sectional studies using the JBI Critical Appraisal Checklist for Analytical Cross-Sectional Studies (*n* = 6).

Study (Author/Year)	Q1	Q2	Q3	Q4	Q5	Q6	Q7	Q8	Overall Risk of Bias
Gabr et al., 2016 [20]	Yes	Yes	Yes	Yes	Unclear	Unclear	Yes	Yes	Low to moderate
Pijnacker et al., 2017 [21]	Unclear	Unclear	Yes	Unclear	Unclear	Unclear	Yes	Yes	Moderate
Wlodarczyk et al., 2018 [22]	Yes	Yes	Yes	Unclear	Unclear	Yes	Yes	Yes	Low to moderate
Kwok et al., 2018 [23]	Unclear	Unclear	Yes	Unclear	Yes	Yes	Yes	Yes	Moderate
Elmahallawi et al., 2022 [24]	Yes	Yes	Yes	Yes	Unclear	Unclear	Yes	Yes	Low to moderate
Barman et al., 2022 [25]	Unclear	Unclear	Yes	Unclear	No	No	Yes	Unclear	High

Note: JBI = Joanna Briggs Institute; Q1 = Inclusion criteria clearly defined; Q2 = Study subjects and setting described in detail; Q3 = Exposure measured in a valid and reliable way; Q4 = Objective, standard criteria used for measurement of the condition; Q5 = Confounding factors identified; Q6 = Strategies to deal with confounding factors stated; Q7 = Outcomes measured in a valid and reliable way; Q8 = Appropriate statistical analysis used. Yes = criterion met; No = criterion not met; Unclear = insufficient information to judge. Overall risk classification: Low to Moderate = ≥6 criteria met with minor gaps; Moderate = 5–6 criteria met with key uncertainty domains; High = ≥2 criteria not met, critically limiting internal validity.

**Table 7:** Methodological quality assessment of Aras et al. [26] using the JBI Critical Appraisal Checklist for Studies Reporting Prevalence Data.

Item	Criterion	Judgement	Rationale/Evidence from the Study
P1	Appropriate sample frame for target population	Yes	The sample comprised children consecutively referred to a tertiary center for electrophysiological assessment due to speech/language pathology—an appropriate clinical frame for characterizing ABR patterns across diagnostic categories, including DLD.
P2	Participants sampled in appropriate way	Unclear	Consecutive clinical referral is implied but not explicitly stated. Referral bias is probable, as children with more severe or atypical presentations are more likely to be referred to for electrophysiological workup.
P3	Sample size adequate (justified)	Unclear	The DLD subgroup comprised 39 children. No priori sample size calculation was reported. Given the small subgroup sizes for some categories (*n* = 15), statistical power for subgroup comparisons is a concern.
P4	Subjects and setting described in detail	Yes	All six diagnostic subgroups are described with sample size, age range, and diagnostic label. Equipment (Eclipse EP 25, Interacoustics) is identified. Clinical and institutional context (Zagreb, Croatia) is stated.
P5	Data analysis with sufficient coverage of sample	Yes	All 123 enrolled participants were included in the analysis. No attrition or missing data reported.
P6	Valid methods for identification of condition (DLD)	Unclear	DLD was diagnosed using the Reynell Developmental Language Scales III (Croatian version), which deviates from CATALISE-2 consensus criteria. Audiological exclusion relied on tympanometry alone—insufficient to rule out mild sensorineural hearing loss.
P7	Condition measured in standard/reliable way	Yes	ABR recorded with Eclipse EP 25 under described stimulus parameters (10 ms duration, 70 dB nHL, 27–65 stimuli/s). ASSR is used as a complementary tool. Measurements applied uniformly across all subgroups.
P8	Appropriate statistical analysis	Yes	Intergroup latency comparisons performed; effects of stimulus rate and age on IPL analyzed. Methods appropriate for the non-comparative observational design.
P9	Response rate adequate (if applicable)	N/A	Not applicable. This is a clinical observational study; no survey or questionnaire was administered, and response rate is not a relevant concept for this design.
Overall	Risk of Bias Classification	Moderate	Of nine appraisal items, four were met (P1, P4, P5, P7, P8), three were unclear (P2, P3, P6), one was not applicable (P9), and none were explicitly not met. The most critical limitation is the absence of a validated DLD diagnosis aligned with CATALISE-2 criteria and the reliance on tympanometry alone for audiological exclusion, which introduces non-trivial risk of diagnostic misclassification. Overall risk is classified as Moderate.

Note. P = prevalence checklist item; Yes = criterion met; Unclear = insufficient information; N/A = not applicable to this study design.

**Table 8:** Certainty of evidence for electrophysiological outcomes in children with Developmental Language Disorder—GRADE approach.

Outcome	No. Studies	Risk of Bias	Inconsistency	Indirectness	Imprecision	Publication Bias	Certainty of Evidence
FFR	3	Serious (−1)	Serious (−1)	Not serious (0)	Serious (−1)	Undetected	LOW
Click-ABR	2	Serious (−1)	Serious (−1)	Not serious (0)	Serious (−1)	Undetected	LOW
LLAEP	2	Some concerns (−0.5)	Not serious (0)	Not serious (0)	Serious (−1)	Undetected	MODERATE
N400	1	Serious (−1)	Serious (−1)	Not serious (0)	Serious (−1)	Undetected	VERY LOW
ASSR	1	Serious (−1)	Serious (−1)	Serious (−1)	Serious (−1)	Undetected	VERY LOW

Note. GRADE = Grading of Recommendations Assessment, Development and Evaluation. All studies are observational; baseline certainly starts at LOW for all outcomes. Downgrade criteria: Risk of bias (serious = −1; some concerns = −0.5); Inconsistency (serious = −1); Indirectness (serious = −1); Imprecision (serious = −1); Publication bias (suspected = −1). Upgrade criteria: large effect (+1/+2); dose–response (+1); plausible confounding would only underestimate the true effect (+1). Certainty levels: High; Moderate; Low. FFR = Frequency Following Response; ABR = Auditory Brainstem Response; LLAEP = Long-Latency Auditory Evoked Potentials; ASSR = Auditory Steady-State Response.

## Data Availability

No new data was created or analyzed in this study. Data sharing is not applicable to this article.

## Supplementary Materials

The following supporting information can be downloaded at: https://www.mdpi.com/article/10.3390/diagnostics16132090/s1, Table S1. PRISMA 2020 Checklist for the reporting of systematic reviews.

## Abbreviations

The following abbreviations are used in this manuscript:

ABR	Auditory Brainstem Response
AEP	Auditory Evoked Potentials
ADHD	Attention-Deficit/Hyperactivity Disorder
AMLR	Auditory Middle Latency Response
ASSR	Auditory Steady State Response Testing
CELF	Clinical Evaluation of Language Fundamentals
CG	Control Group
CAEP	Cortical Auditory Evoked Potentials
CATALISE	Multinational and Multidisciplinary Delphi Consensus Study
DeCS	Health Sciences Descriptors
DLD	Developmental Language Disorder
DSM-5-TR	Diagnostic and Statistical Manual of Mental Disorders, Fifth Edition, Text Revision
EEG	Electroencephalography
ERP	Event-Related Potentials
FFR	Frequency Following Response
GRADE	Certainty of Evidence Synthesis
HPF	High-Pass Filter
ICC	Intraclass Correlation Calculation
IHS	Intelligent Hearing System
JBI	Joanna Briggs Institute
LPF	Low-Pass Filter
LLAEP	Long-Latency Auditory Evoked Potentials
MeSH	Medical Subject Headings
OME	Otitis Media with Effusion
PICOT	Population, Intervention, Comparison, Outcome, and Time
PLS	Preschool Language Scale
PPVT	Peabody Picture Vocabulary Test
PRISMA	Preferred Reporting Items for Systematic Reviews and Meta-Analyses
PTA	Pure Tone Audiometry
QCRI	Qatar Computing Research Institute
SCALES	Studying Communication in Language and Education
SD	Standard Deviation
Stim. Duration	Stimulus Duration
SG	Study Group
SLI	Specific Language Impairment
SPL	Sound Pressure Level
STT-II	Schlichting Test voor Taalproductie-ii
STvT	Schlichting Test for Language Comprehension
SWiM	Synthesis Without Meta-analysis
WNV	Wechsler Nonverbal Scale of Ability
WASI	Wechsler Abbreviated Intelligence Scale
AW	Analysis Window
BF	Bandpass Filter
NR	Not Reported
P	Polarity
PR	Presentation Rate
S	Sweeps
SI	Stimulus Intensity
dB nHL	decibels Hearing Level
dB SPL	decibels Sound Pressure Level
