# Supplementary material for: Auditory Electrophysiological Findings in Children with Developmental Language Disorder: A Systematic Review
Source: Diagnostics (Basel). 2026 Jul 3;16(13):2090. doi: 10.3390/diagnostics16132090 (PMC13361366; doi:10.3390/diagnostics16132090)
Supplement: Supplementary file 1 [file diagnostics-16-02090-s001.zip › diagnostics-4359917-supplementary.pdf]

Table S1. PRISMA 2020 Checklist for the reporting of systematic reviews (Page MJ et al. BMJ 2021;372:n71).

Section and Topic	Item #	Checklist item	Location where item is reported
**TITLE**			
Title	1	Identify the report as a systematic review.	Title page, Line 01
**ABSTRACT**			
Abstract	2	See the PRISMA 2020 for Abstracts checklist.	Abstract session, Lines 15-29
**INTRODUCTION**			
Rationale	3	Describe the rationale for the review in the context of existing knowledge.	Section 1, Lines 35-45
Objectives	4	Provide an explicit statement of the objective(s) or question(s) the review addresses.	Section 1, Lines 45; 55-59
**METHODS**			
Eligibility criteria	5	Specify the inclusion and exclusion criteria for the review and how studies were grouped for the syntheses.	Section 2.1, Lines 61–83
Information sources	6	Specify all databases, registers, websites, organisations, reference lists and other sources searched or consulted to identify studies. Specify the date when each source was last searched or consulted.	Section 2.2., Line 87
Search strategy	7	Present the full search strategies for all databases, registers and websites, including any filters and limits used.	Section 2.2., Lines 87–97
Selection process	8	Specify the methods used to decide whether a study met the inclusion criteria of the review, including how many reviewers screened each record and each report retrieved, whether they worked independently, and if applicable, details of automation tools used in the process.	Section 2.3., Lines 99–101
Data collection process	9	Specify the methods used to collect data from reports, including how many reviewers collected data from each report, whether they worked independently, any processes for obtaining or confirming data from study investigators, and if applicable, details of automation tools used in the process.	Section 2.4., Lines 103–105<br>Tables 3–4 (lines 111–136)
Data items	10a	List and define all outcomes for which data were sought. Specify whether all results that were compatible with each outcome domain in each study were sought (e.g. for all measures, time points, analyses), and if not, the methods used to decide which results to collect.	Section 2.4, Lines 103–105<br>Table 1 (lines 55–59)
	10b	List and define all other variables for which data were sought (e.g. participant and intervention characteristics, funding sources). Describe any assumptions made about any missing or unclear information.	Section 2.4, Lines 103–105<br>Tables 3–4 (lines 111–136)
Study risk of bias assessment	11	Specify the methods used to assess risk of bias in the included studies, including details of the tool(s) used, how many reviewers assessed each study and whether they worked independently, and if applicable, details of automation tools used in the process.	Section 3.2, Lines 146–148<br>Table 5A, lines 150–161)<br>Table 5B, lines 165–180)
Effect measures	12	Specify for each outcome the effect measure(s) (e.g. risk ratio, mean difference) used in the synthesis or presentation of results.	Section 2.4, Lines 107–108
Synthesis	13a	Describe the processes used to decide which studies were eligible for each synthesis (e.g. tabulating the study	Section 2.4, Lines 107–108

Section and Topic	Item #	Checklist item	Location where item is reported
methods		intervention characteristics and comparing against the planned groups for each synthesis (item #5)).	Section 3.5, Lines 208–262<br>Section 2.1, lines 61–83
	13b	Describe any methods required to prepare the data for presentation or synthesis, such as handling of missing summary statistics, or data conversions.	Section 2.4, Lines 103–105<br>Tables 3–4, Lines 111–136
	13c	Describe any methods used to tabulate or visually display results of individual studies and syntheses.	Section 2.4, Lines 103–105<br>Table 3 (lines 111–123)<br>Table 4 (lines 125–136)<br>Sections 3.5.1–3.5.5 (lines 212–262)
	13d	Describe any methods used to synthesize results and provide a rationale for the choice(s). If meta-analysis was performed, describe the model(s), method(s) to identify the presence and extent of statistical heterogeneity, and software package(s) used.	Section 2.4 · Lines 107–108
	13e	Describe any methods used to explore possible causes of heterogeneity among study results (e.g. subgroup analysis, meta-regression).	N/A — Formal heterogeneity analyses not conducted given small number of studies per outcome and narrative design; sources of heterogeneity discussed qualitatively in Section 4, lines 282–338
	13f	Describe any sensitivity analyses conducted to assess robustness of the synthesized results.	N/A — Sensitivity analyses not performed due to small number of included studies (n=7) and narrative SWiM design; acknowledged as limitation in Section 4.7, Line 338
Reporting bias assessment	14	Describe any methods used to assess risk of bias due to missing results in a synthesis (arising from reporting biases).	Section 2.4, Line 107<br>Section 4.7, Line 338
Certainty assessment	15	Describe any methods used to assess certainty (or confidence) in the body of evidence for an outcome.	Section 3.6, Lines 264–278<br>Table 6 (lines 268–278)
**RESULTS**			
Study selection	16a	Describe the results of the search and selection process, from the number of records identified in the search to the number of studies included in the review, ideally using a flow diagram.	Section 3.1,Lines 140–144
	16b	Cite studies that might appear to meet the inclusion criteria, but which were excluded, and explain why they were excluded.	Section 3.1, Line 144
Study	17	Cite each included study and present its characteristics.	Table 3, Lines 111–123

Section and Topic	Item #	Checklist item	Location where item is reported
characteristics			Table 4, Lines 125–136
Risk of bias in studies	18	Present assessments of risk of bias for each included study.	Section 3.2, Lines 146–180
Results of individual studies	19	For all outcomes, present, for each study: (a) summary statistics for each group (where appropriate) and (b) an effect estimate and its precision (e.g. confidence/credible interval), ideally using structured tables or plots.	Table 3, Lines 111–123<br>Sections 3.5.1–3.5.5, Lines 212–262
Results of syntheses	20a	For each synthesis, briefly summarise the characteristics and risk of bias among contributing studies.	Sections 3.5.1–3.5.5, Lines 212–262<br>Table 6, Lines 268–278
	20b	Present results of all statistical syntheses conducted. If meta-analysis was done, present for each the summary estimate and its precision (e.g. confidence/credible interval) and measures of statistical heterogeneity. If comparing groups, describe the direction of the effect.	N/A — No meta-analysis performed; narrative synthesis results presented in sections 3.5.1–3.5.5 (lines 212–262) with direction of effects described qualitatively.
	20c	Present results of all investigations of possible causes of heterogeneity among study results.	Section 4, Lines 282–338;107, 190
	20d	Present results of all sensitivity analyses conducted to assess the robustness of the synthesized results.	N/A — No sensitivity analyses conducted (see item 13f).<br>Section 4.7, Line 338
Reporting biases	21	Present assessments of risk of bias due to missing results (arising from reporting biases) for each synthesis assessed.	Table 6, Lines 268–278<br>Section 4.7, Line 338
Certainty of evidence	22	Present assessments of certainty (or confidence) in the body of evidence for each outcome assessed.	Section 3.6 and Table 6, Lines 264–278<br>Section 4.6, Lines 332–334
**DISCUSSION**			
Discussion	23a	Provide a general interpretation of the results in the context of other evidence.	Section 4, Lines 282–326
	23b	Discuss any limitations of the evidence included in the review.	Section 4.5, Lines 328–330<br>Section 4.6, Lines 332–334
	23c	Discuss any limitations of the review processes used.	Section 4.7, Lines 336–338
	23d	Discuss implications of the results for practice, policy, and future research.	Lines 340–342
**OTHER INFORMATION**			
Registration and protocol	24a	Provide registration information for the review, including register name and registration number, or state that the review was not registered.	Section 2, Lines 49, 29.

Section and Topic	Item #	Checklist item	Location where item is reported
	24b	Indicate where the review protocol can be accessed, or state that a protocol was not prepared.	Section 2, Lines 49, 61–83
	24c	Describe and explain any amendments to information provided at registration or in the protocol.	N/A — Review was not registered and no formal protocol was prepared (see item 24a)
Support	25	Describe sources of financial or non-financial support for the review, and the role of the funders or sponsors in the review.	Lines 348, 27
Competing interests	26	Declare any competing interests of review authors.	Line 356
Availability of data, code and other materials	27	Report which of the following are publicly available and where they can be found: template data collection forms; data extracted from included studies; data used for all analyses; analytic code; any other materials used in the review.	Line 354

From: Page MJ, McKenzie JE, Bossuyt PM, Boutron I, Hoffmann TC, Mulrow CD, et al. The PRISMA 2020 statement: an updated guideline for reporting systematic reviews. BMJ 2021;372:n71. doi: 10.1136/bmj.n71. This work is licensed under CC BY 4.0. To view a copy of this license, visit https://creativecommons.org/licenses/by/4.0/

Note: Cells shaded yellow (N/A) indicate items not applicable to this review due to its narrative SWiM design, unregistered protocol, and absence of meta-analysis. All applicable PRISMA 2020 items are reported in the manuscript.
